# In situ elemental analyses of living biological specimens using ‘NanoSuit’ and EDS methods in FE-SEM

**DOI:** 10.1038/s41598-020-71523-8

**Published:** 2020-09-03

**Authors:** Yasuharu Takaku, Sayuri Takehara, Chiaki Suzuki, Hiroshi Suzuki, Masatsugu Shimomura, Takahiko Hariyama

**Affiliations:** 1 Preeminent Medical Photonics Education and Research Center, Institute for NanoSuit Research, Hamamatsu University School of Medicine, 1-20-1 Handayama, Higashi-ku, Hamamatsu, 431-3192 Japan; 2 Department of Chemistry, Hamamatsu University School of Medicine, 1-20-1 Handayama, Higashi-ku, Hamamatsu, 431-3192 Japan; 3 Department of Bio- and Material Photonics, Chitose Institute of Science and Technology, 758-65, Chitose, Hokkaido 066-8655 Japan

**Keywords:** Electron microscopy, Optical imaging

## Abstract

Energy dispersive X-ray spectroscopy (EDS) carried out alongside scanning electron microscopy (SEM) is a common technique for elemental analysis. To investigate “wet” biological specimens, complex pre-treatments are required to stabilize them under the high vacuum conditions of high-resolution SEM. These often produce unwanted artifacts. We have previously reported that the polymerization of natural surface substances on organisms by the electron beam of the SEM setup or by plasma irradiation causes a nano-scale layer to form—called a “NanoSuit”—that can act as a barrier and keep organisms alive and hydrated in a field-emission SEM system. In the study reported herein, we examined the suitability of the NanoSuit method for elemental analyses of biological specimens by EDS. We compared experimental results for living *Drosophila* larvae and *Aloe arborescens* specimens prepared by the NanoSuit method and by conventional fixation. The NanoSuit method allowed accurate detection of the elemental compositions at high resolution. By contrast, specimens prepared by the conventional fixation method displayed additional EDS signals corresponding to the elements in the chemicals involved in the fixation process. Our results demonstrate that the NanoSuit method is useful for studying hydrous samples via EDS and SEM, particularly in biological sciences.

## Introduction

When a sample is irradiated by an electron beam under high vacuum conditions in a scanning electron microscope (SEM), characteristic X-rays are generated. These X-rays can be used to analyze the elemental composition of the materials on the micro- or nano-scale using an energy dispersive X-ray spectroscopy (EDS) detector^1^. EDS is a commonly used technique to analyze stable inorganic materials, such as metals and minerals. Biological specimens, however, because of their high water content require extensive sample preparation to withstand the high vacuum conditions in the SEM and to permit electrical conduction through the biopolymers. These conventional preparation methods involve complex pre-treatments of the biological samples such as chemical fixation, dehydration, freeze-drying or critical point drying, and metal coating^2^. Therefore, these procedures preclude the direct observation of living specimens. Furthermore, the evaporation of water from living organisms often produces unwanted artifacts even for fixed specimens. Moreover, such fixation procedures require treatment of the samples by solutions, which may result in alterations in the localisation of elements in the sample. Thus, the currently available EDS methods for biological specimens may not be adequate to reveal the in vivo spatial distributions of elements.

Previously, we reported that a simple surface modification of living samples forms a nano-scale surface layer—which we call a “NanoSuit”—that prevents gases and liquids present in small animals from evaporating under the high vacuum conditions in a field emission-scanning electron microscope (FE-SEM)^3–5^. We found that when natural extracellular substances (ECS), which cover the surfaces of some animals, are polymerized by plasma or electron beam irradiation, the polymerized membrane (NanoSuit) renders the organisms extremely tolerant to high vacuum conditions^3,4^. We also found that, in some cases, for animals with no evident natural ECS, various monomer solutions can mimic the ECS, functioning as a substitute ECS when added to the surface of specimens and polymerized^3–6^. Furthermore, we have shown that plant surface substances act in a similar manner to the animal ECS when exposed to electron beam irradiation^7,8^. In these investigations, we found that, when compared to specimens prepared by conventional fixation methods, the surface fine structure of organisms covered with a NanoSuit was intact and well preserved. The technique enabled high-resolution imaging and observation of biological samples at magnifications exceeding 20,000×^9^. The present study further explores the specific properties of the NanoSuit and examines its suitability for elemental analysis in biological specimens. Here, we show that EDS can successfully and accurately detect in situ elemental compositions of hydrous biological specimens coated with the NanoSuit.

## Results and discussion

### NanoSuit plays a significant role as a barrier to reduce the effects of high vacuums

**Figure 1:**
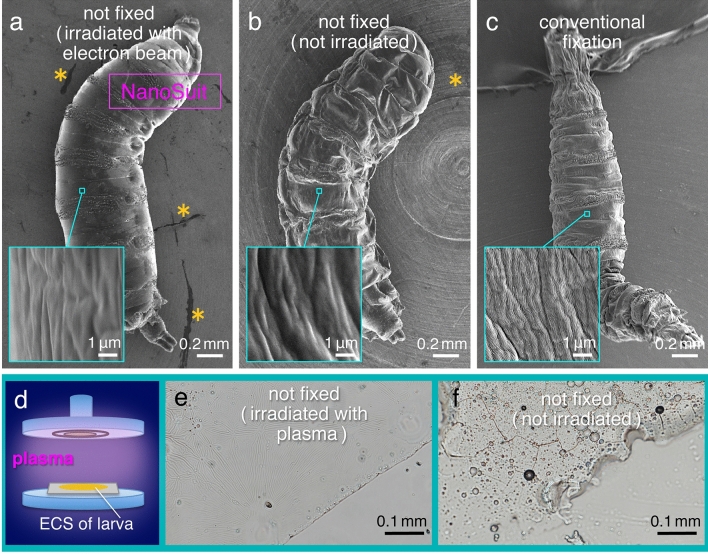
Comparison of the SEM images for three types of sample preparations. (**a**) Living larva of *Drosophila* exposed to high vacuum with electron beam irradiation for 60 min. (**b**) Living larva placed in the observation chamber without electron beam irradiation for 60 min prior to SEM observation. (**c**) Specimen prepared by conventional fixation methods for SEM. (**d**) Schematic illustration of the plasma irradiation of the extra cellular substances (ECS) extracted from the surface of *Drosophila* larvae (see “Methods”). (**e**) Light microscopy (LM) image of newly synthesized membrane following plasma polymerization. (**f**) Without plasma irradiation, the membrane was not formed as in the process shown in (**e**). Asterisks in (**a**,**b**) indicate the positions of ECS that rubbed off on the observation stub during the movements of the larvae (seen in dark gray).

**Figure 2:**
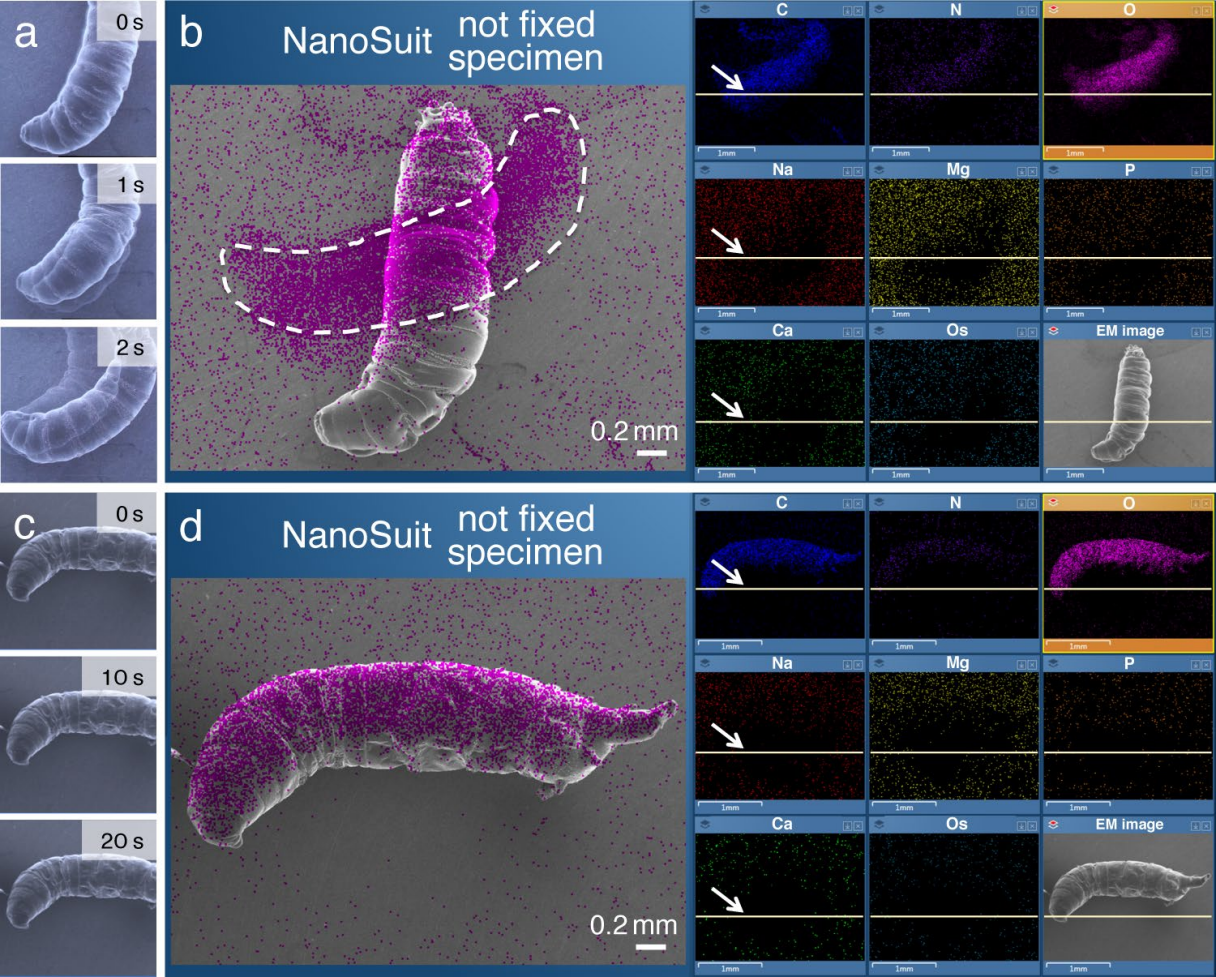
(**a**,**c**) Sequential SEM images of living larvae of *Drosophila* that underwent electron beam irradiation immediately before the SEM observation (the NanoSuit method). The blurs in (**a**) are interpreted as the evidence for active movements. (**b**,**d**) EDS mapping analysis. The magenta signal indicates the localization of elemental oxygen. Note that the larger area covered by elemental oxygen in (**b**) is due to the movement (displacement) of the living larva during the measurements. The arrows in (**b**,**d**) indicate the current positions of the scanning electron beam (cf. Supplementary movie 4).

We have previously reported that at atmospheric pressure the surfaces of *Drosophila* larvae are evidently covered in natural ECS (Supplementary movie 1)^3^, which suggested that ECS might be components of the NanoSuit. In the present investigation, the morphology of *Drosophila* larvae in SEM images acquired under the same vacuum conditions were compared for the following three different sample preparation methods: (1) living specimens underwent electron beam irradiation for 60 min (Fig. 1a); (2) living specimens were placed in the SEM observation chamber for 60 min without concurrent electron beam irradiation (Fig. 1b); and (3) specimens were prepared by conventional fixation methods for SEM (Fig. 1c). Our results showed that the overall morphology of specimens irradiated with the electron beam was well preserved during SEM image acquisition (Fig. 1a). By comparison, the untreated samples (high vacuum but no irradiation) (Fig. 1b) and the conventionally fixed samples (Fig. 1c) had poorly preserved morphology. These results confirm previous observations that electron beam irradiation in the SEM setup leads to the formation of a thin layer (NanoSuit) on the surface of living larvae, which protects them from the high vacuum SEM conditions^3–5,9^.

Previously, we reported that the NanoSuit can be formed immediately by electron beam irradiation. Thus, we can observe living specimens soon after their irradiation in an SEM instrument (cf. Fig. 2)^3–5^. Moreover, as the NanoSuit is very thin (10–100 nm)^3,9^, the electron beam can penetrate it and reveal the fine structures of the surface underneath it; in other words, the NanoSuit does not interfere with high resolution SEM imaging^5,9^. In the present study, we investigated the formation of the NanoSuit in vitro by demonstrating the polymerization of the ECS (Supplementary movie 1) as a self-standing membrane (Fig. 1d) (see “Methods”)^3,6^. ECS collected from larvae formed a membranous structure when irradiated by plasma. This structure was insoluble in water and clearly showed the effects of plasma polymerization after irradiation (Fig. 1e; Supplementary movie 2). In contrast, ECS that had not been treated by plasma irradiation did not form such a membranous structure and changed into a semi-dried substance (Fig. 1f) that was soluble in water (Supplementary movie 3).

### NanoSuit enables in situ elemental measurements for living specimens in the FE-SEM

The NanoSuit technique allows small animals to make spontaneous movements during SEM observation^3–5^. Although the imaging system of a conventional SEM system is designed for motionless objects, the ‘video mode’ can be used to observe the active movement of living animals (see “Methods”). However, EDS requires multistage scanning for each measurement to achieve high resolution analysis; therefore, a dynamic EDS analysis has been impossible until now. If the specimen moves during the measurement, a blur appears in the acquired image (Fig. 2a,b; Supplementary movie 4). Due to this limitation, EDS analyses were successfully performed only when the live animals were motionless (Fig. 2c,d).

**Figure 3:**
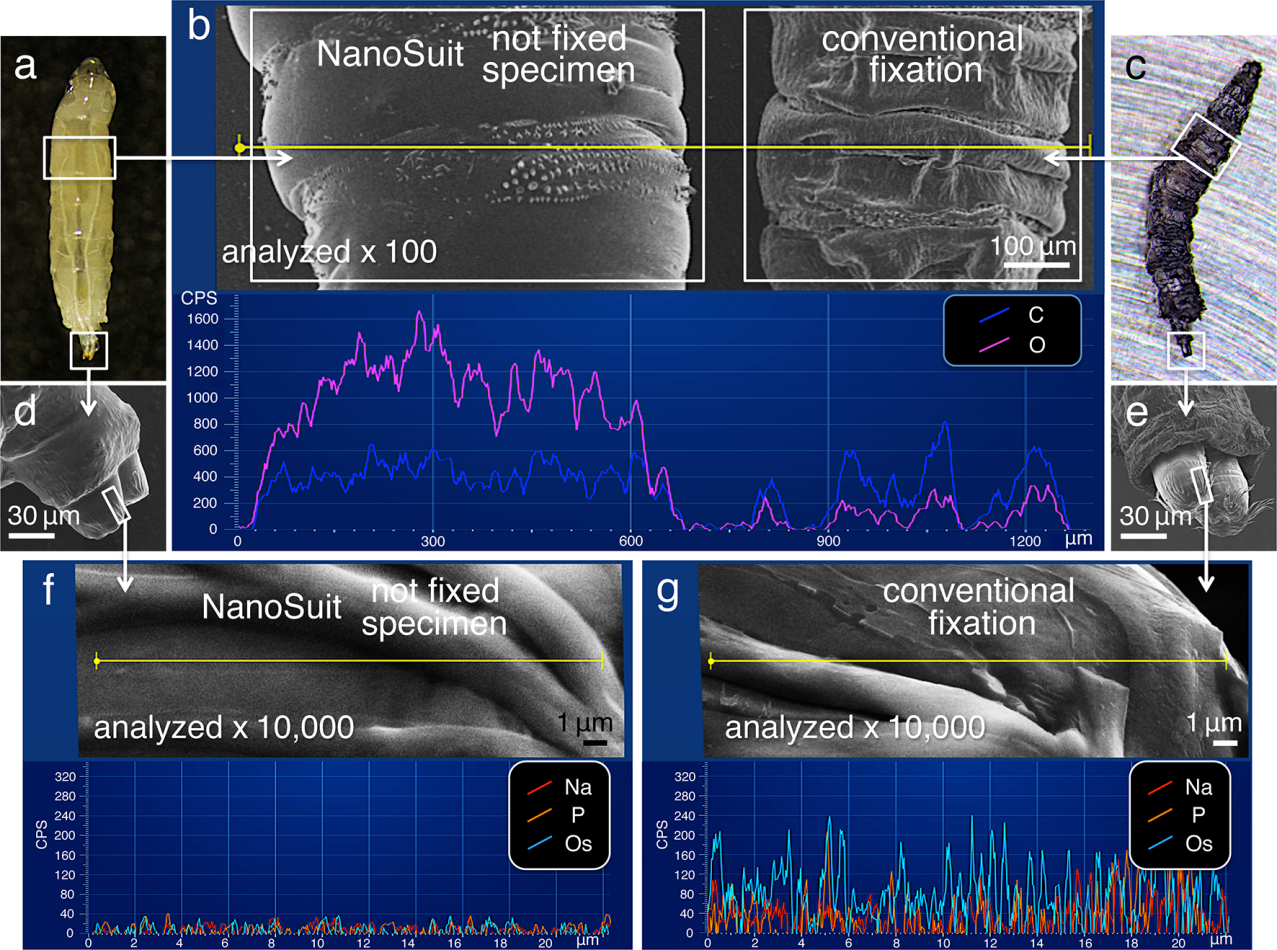
Comparison of light microscopy (LM) images of a living *Drosophila* larva (**a**) and a specimen prepared by conventional fixation methods for SEM (**c**). Comparison of low magnification SEM images of the surfaces of specimens (**b**) and high magnification SEM images of spiracle tips (**d**–**g**). EDS line scan analysis (along the yellow line) in the two types of sample preparations (**b**,**f**,**g**) is plotted below in the same panels. Carbon (C; blue), oxygen (O; magenta), sodium (Na; red), phosphorus (P; orange), and osmium (Os; light blue).

The EDS analysis of representative elements in specimens showed that the oxygen peak was significantly higher in the specimens protected with the NanoSuit (1,056 ± 345 counts per second (CPS); mean ± SD, *N* = 20) compared with those prepared using conventional fixation methods (122 ± 134 CPS; mean ± SD, *N* = 20) (*P* < 0.01) (Fig. 3a–c; Supplementary Fig. S1a; Table 1). Since the water content of living organisms is approximately 70–80%, the high amount of oxygen is expected to originate from the water content in the hydrous specimens protected with the NanoSuit (Fig. 3a,b), whereas water is removed during the drying process in the specimens prepared by conventional fixation (Fig. 3b,c).

**Table 1:** Average peak counts (counts per second) for the elemental signals from EDS line scans of larvae specimens and *Aloe* samples protected with the NanoSuit or prepared by the conventional fixation method (mean ± SD, *N* = 20).

Element	NanoSuit method	Conventional fixation
*Drosophila* larva		
O	1,056.1 ± 345.7	122.1 ± 134.4
Na	27.7 ± 30.5	41.7 ± 38.1
P	32.3 ± 36.3	67.2 ± 55.5
Os	12.7 ± 9.0	145.4 ± 121.5
*Aloe*		
Ca	229.4 ± 95.8	238.7 ± 106.7
Na	17.5 ± 11.9	214.7 ± 84.5
P	16.9 ± 13.3	384.6 ± 123.9
Os	12.1 ± 6.7	271.3 ± 60.2

Next, we compared the elemental compositions of specimens protected with the NanoSuit (Fig. 3d,f) and those prepared by conventional fixation methods (Fig. 3e,g) at magnifications exceeding 10,000×. Such measurements cannot be made by low-vacuum SEM (Lv-SEM) or by environmental SEM (E-SEM) because the reduced vacuum conditions used in these systems result in inferior resolution, even with a maximum magnification of 2,000×^10,11^. In the present investigation, specimens protected with the NanoSuit exhibited weak elemental signals for sodium and phosphorus (Fig. 3f; Table 1), while specimens prepared with conventional fixation methods showed significantly stronger sodium and phosphorus peaks (*P* < 0.01) (Fig. 3g; Table 1). These elements are derived from the sodium phosphate buffer used in the sample preparation process (Supplementary Fig. S2a). The polymerized thin membrane formed from the ECS of the specimens (NanoSuit) did not generate such significant sodium and phosphorus EDS peaks, which supports our claims regarding the suitability of this method (Supplementary Fig. S2b). As expected, high intensity osmium peaks were also measured in the specimens prepared via the conventional methods (Fig. 3g; Supplementary Fig. S1c), which use osmium for post-fixing and the surface coating of specimens (see “Methods”). If the specimens are not coated with an electrically conductive material, such as osmium, gold, palladium, or platinum^2^, the samples always show electrostatic charging, precluding satisfactory imaging during SEM observation^5^.

**Figure 4:**
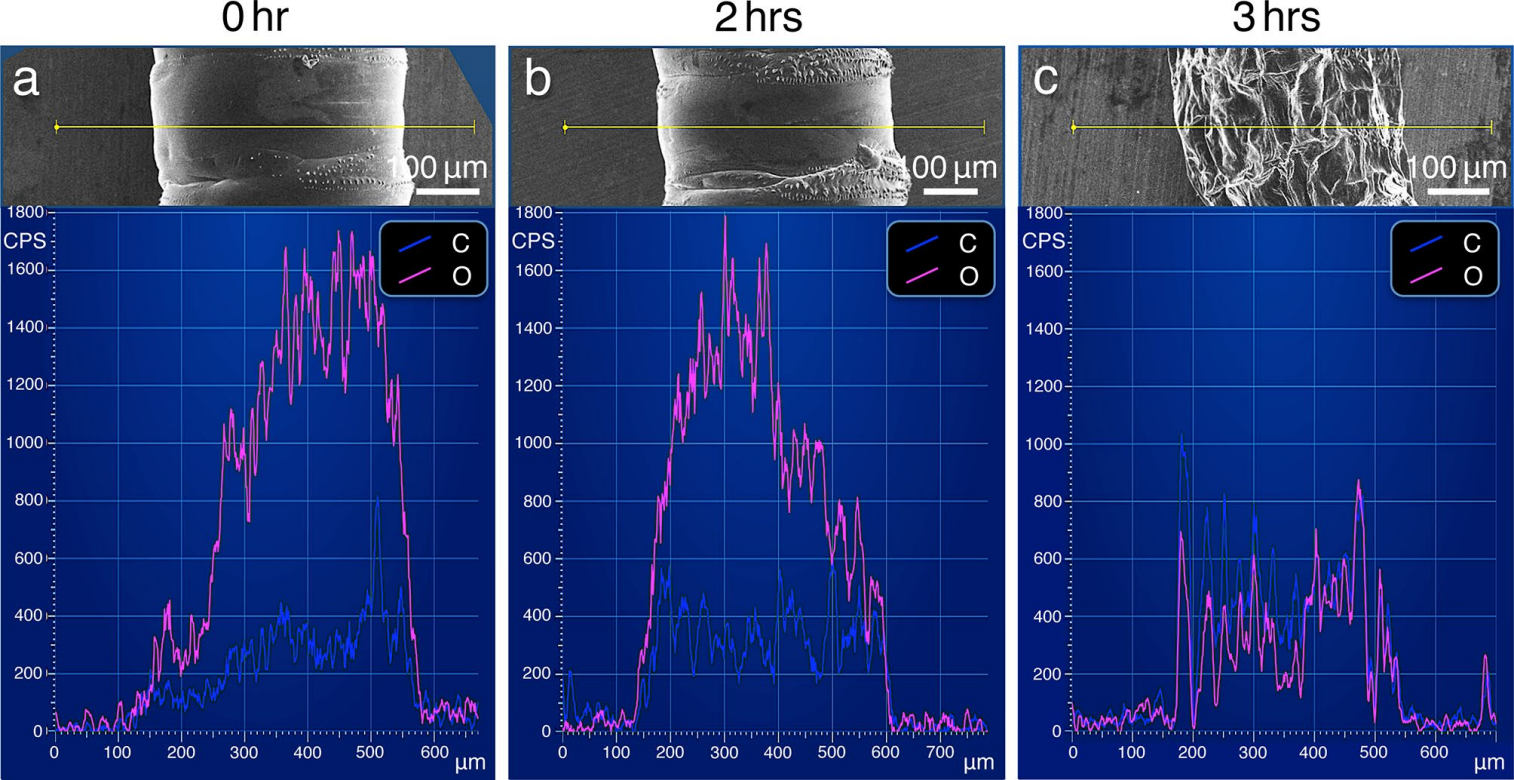
Comparison of the EDS line scan analysis (along the yellow line) for a *Drosophila* larva prepared with the NanoSuit method at 0, 2 and 3 h. Carbon (C; blue), oxygen (O; magenta).

In contrast, the overall morphology of the NanoSuit specimens was well preserved and showed no electrostatic charging during 120 min of continuous observation (Figs. 3b,d,f, 4a,b). Even after the cessation of active movement in the SEM, when atmospheric pressure was restored to the environment of the larvae, the animals recovered movement within a few minutes, indicating that the specimens were maintained alive during the SEM observation period. Nevertheless, when the data collection time for EDS analysis was extended to 180 min, the surfaces of the larvae started to shrink due to desiccation, and the specimens showed electrostatic charging (Fig. 4c, inset). For these measurements, the oxygen peak was reduced in intensity (318 ± 194 CPS; mean ± SD, *N* = 20) (Fig. 4c; Supplementary Fig. S1b), suggesting a decrease in water content. We have previously reported that specimens protected with the NanoSuit did not show electrostatic charging, as long as the specimens were alive^4,5^. Moreover, in the experiments involving dead specimens, even though it was possible to form NanoSuits on their surfaces in the SEM instrument, an immediate accumulation of electrostatic charge on the surface was evident from the subsequent SEM observations^5^. These results suggest that living organisms possess electrical conductors and/or are endowed with charge-free properties on their surface. Furthermore, based on the present investigation, it is also suggested that water contained inside the living organisms plays a role in inhibiting electrostatic charging. The NanoSuit seems to prolong the survival time of living specimens under vacuum and charge-free conditions.

### The plant NanoSuit plays a significant role as a barrier preventing desiccation

**Figure 5:**
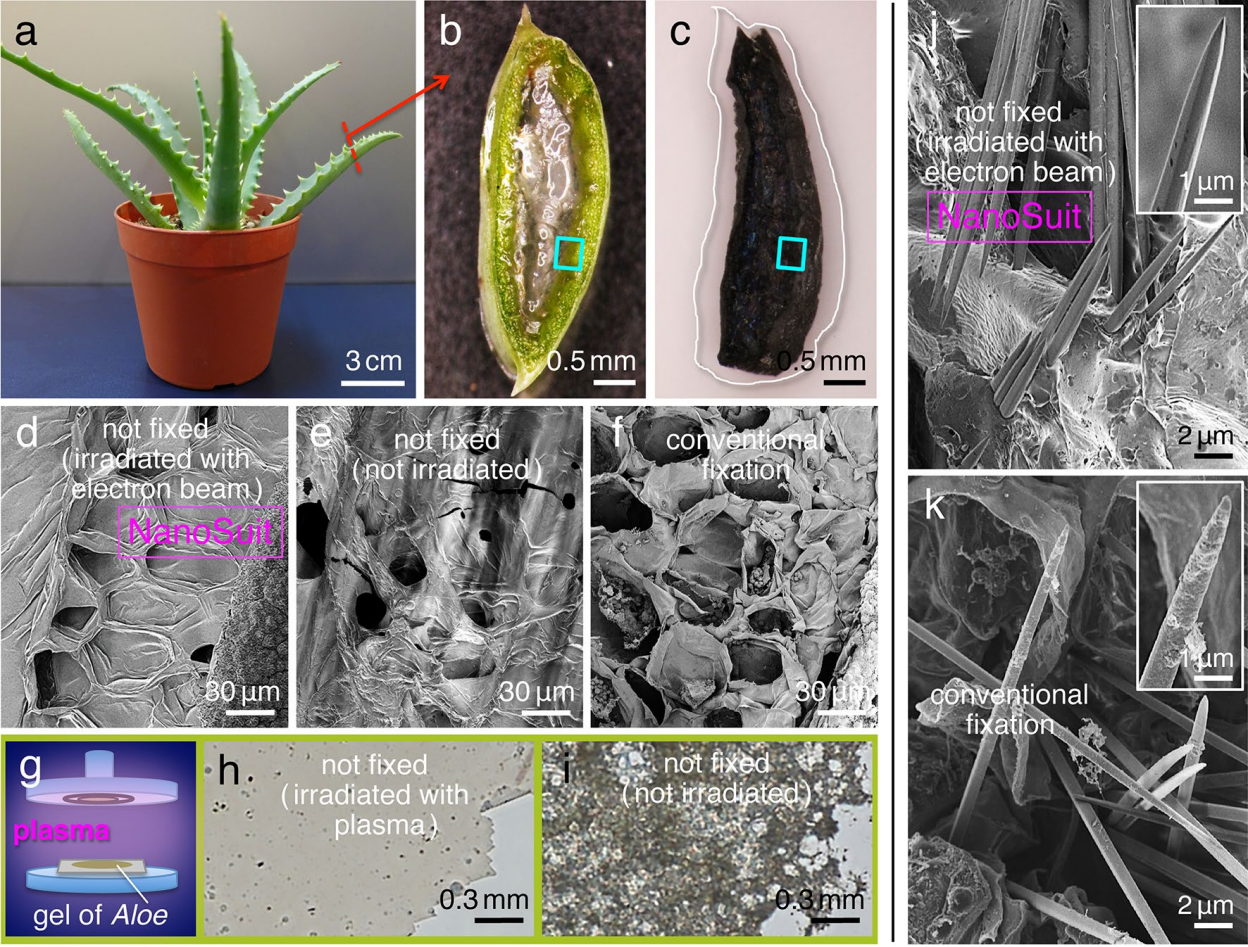
(**a**) *Aloe arborescens* plant. Comparison of light microscopy (LM) images of a cross-sectioned fresh *Aloe* leaf slice (**b**) and another prepared by conventional fixation methods for SEM (**c**). The white line surrounding the darkened leaf slice in (**c**) indicates the original shape of the specimen before conventional fixation and sample preparation. The rectangles in (**b**,**c**) indicate the region analyzed by FE-SEM in the present experiments. (**d**–**f**) Comparison of SEM images of the surface of the leaf slice. (**d**) The fresh hydrous specimen was exposed to high vacuum with electron beam irradiation for 30 min. (**e**) Prior to SEM observation, a fresh specimen was placed in the observation chamber without electron beam irradiation for 30 min. The specimen showed electrostatic charging when subsequently observed by SEM. (**f**) A specimen prepared by conventional fixation methods for SEM with an osmium coating on the surface to avoid electrostatic charging. (**g**) Schematic illustration of the plasma irradiation of the gel extracted from the interior of the *Aloe* leaf (see “Methods”). (**h**) LM image of newly synthesized membrane following plasma polymerization. (**i**) Without plasma irradiation, the membrane observed in (**h**) was not formed. Comparison of the SEM images of the needle-like calcium oxalate crystals in a cross-sectioned leaf slice prepared by NanoSuit method (**j**) and conventional fixation method (**k**).

To examine whether the new NanoSuit method can also be applied to plants, we chose *Aloe arborescens* (referred to as *Aloe* hereafter) as a specimen in the present investigations (Fig. 5a). A particularly useful feature of *Aloe* for examining the response to a high vacuum environment is that the plant can tolerate desiccation. This property is mainly as result of the gel-filled matrix of the plant, which consist of anthraquinones, polysaccharides, and low-molecular-weight substances (Fig. 5b; Supplementary movie 5)^12^. The *Aloe* gel is used in various foods, cosmetics, and medical products as a main ingredient^13,14^.

SEM images of cross-sectioned *Aloe* leaf slices were compared for specimens prepared with three different methods similar to those used for animal specimens (Fig. 5b–f). Our results showed that only specimens irradiated with the electron beam were well preserved in the SEM (Fig. 5d). Non-irradiated specimens were not protected from desiccation under high vacuum conditions. This suggests that the properties of the *Aloe* gel were not sufficient to stabilize the matrix to resist the effects of high vacuum SEM. The NanoSuit, however, formed a polymerized membrane around the *Aloe* leaves with the assistance of energy from the electron beam, providing a barrier to desiccation. Experimental evidence for the formation of membranous structures by plasma irradiation of gel materials from the *Aloe* leaf also supports this concept (Fig. 5g–i; Supplementary movies 6, 7)^7^.

### NanoSuit permits in situ elemental analysis for hydrous plant specimens in the FE-SEM instrument

A second important advantage of *Aloe* is that needle-like calcium oxalate crystals are naturally formed inside leaves^15^, suggesting that calcium should be detectable in the crystals. To examine the needle-like calcium oxalate crystals by FE-SEM, morphological features observed via the NanoSuit and conventional fixation methods were compared (Fig. 5j,k). The SEM images of specimens protected with the NanoSuit displayed well-preserved needle-like crystals on each cross-sectioned surface (565 ± 39; mean ± SD, *N* = 20), whereas the number of the crystals was significantly lower (254 ± 19; mean ± SD, *N* = 20) (*P* < 0.01) in the specimens prepared by the conventional fixation method. In some of the latter cases, the surfaces of the crystals were covered with shreds of extra material that was not present with on the samples prepared via the NanoSuit method (insets in Fig. 5j,k). These extra fragments of material appearing in the specimens prepared by the conventional method could be a byproduct of some aspect of the multi-step sample preparation process.

**Figure 6:**
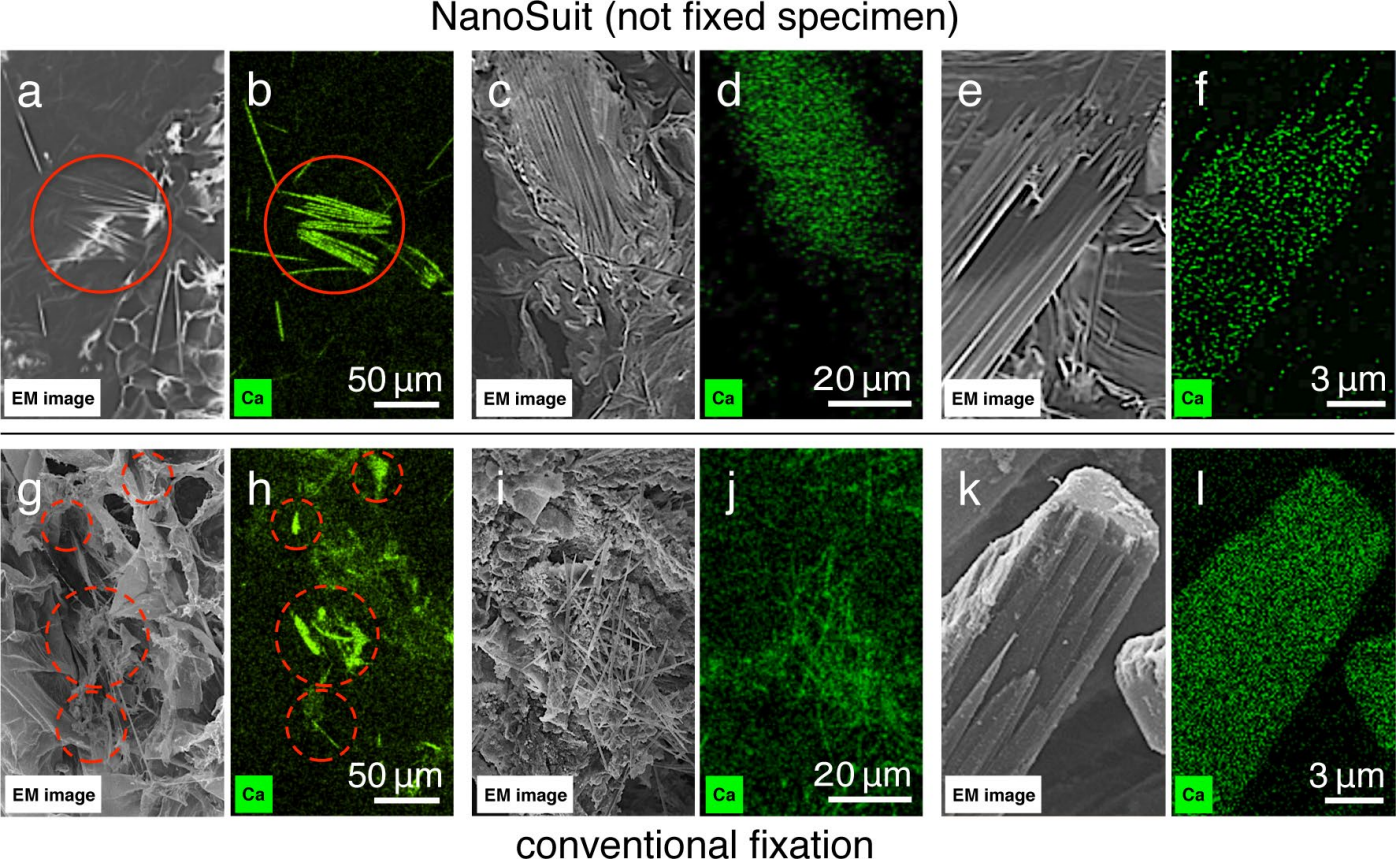
Low magnification and high magnification SEM images of an *Aloe* leaf slice prepared by the NanoSuit method (**a**,**c**,**e**) and by the conventional fixation method (**g**,**i**,**k**). The EDS calcium maps are shown in (**b**,**d**,**f**,**h**,**j**,**l**) with the corresponding SEM images in (**a**,**c**,**e**,**g**,**i**,**k**). Green signals indicate the localization of elemental calcium, which was clearly detected on the needle-like crystals in specimens prepared with the NanoSuit method (red circles in **a**,**b**). In specimens prepared via the conventional fixation method, strong calcium signals were identified at the locations of needle-like crystals (red dotted circles in **g**,**h**); however, elemental Ca was also located in other areas that did not contain needle-like crystals.

To compare the EDS results for specimens prepared with the NanoSuit with those for the samples prepared by conventional fixation methods, we created maps of the calcium content. High magnification EDS measurements of the NanoSuit specimens clearly showed that calcium was localized on the needle-like crystals (Fig. 6a–f). By comparison, in the conventionally fixed samples, calcium was not only detected on the needle-like crystals but also on other structures (Fig. 6g,h). In some areas, calcium was observed on and around broken crystals (Fig. 6i,j) as well as in thick bundles formed by the aggregation of crystal needles (Fig. 6k,l). Hence, in the specimens prepared by the conventional fixation method, it is difficult to determine whether the observed calcium signal originates from the needle-shaped structures or other structures. Such a broad distribution of elemental calcium could be a result of tissue damage during the sample preparation process.

**Figure 7:**
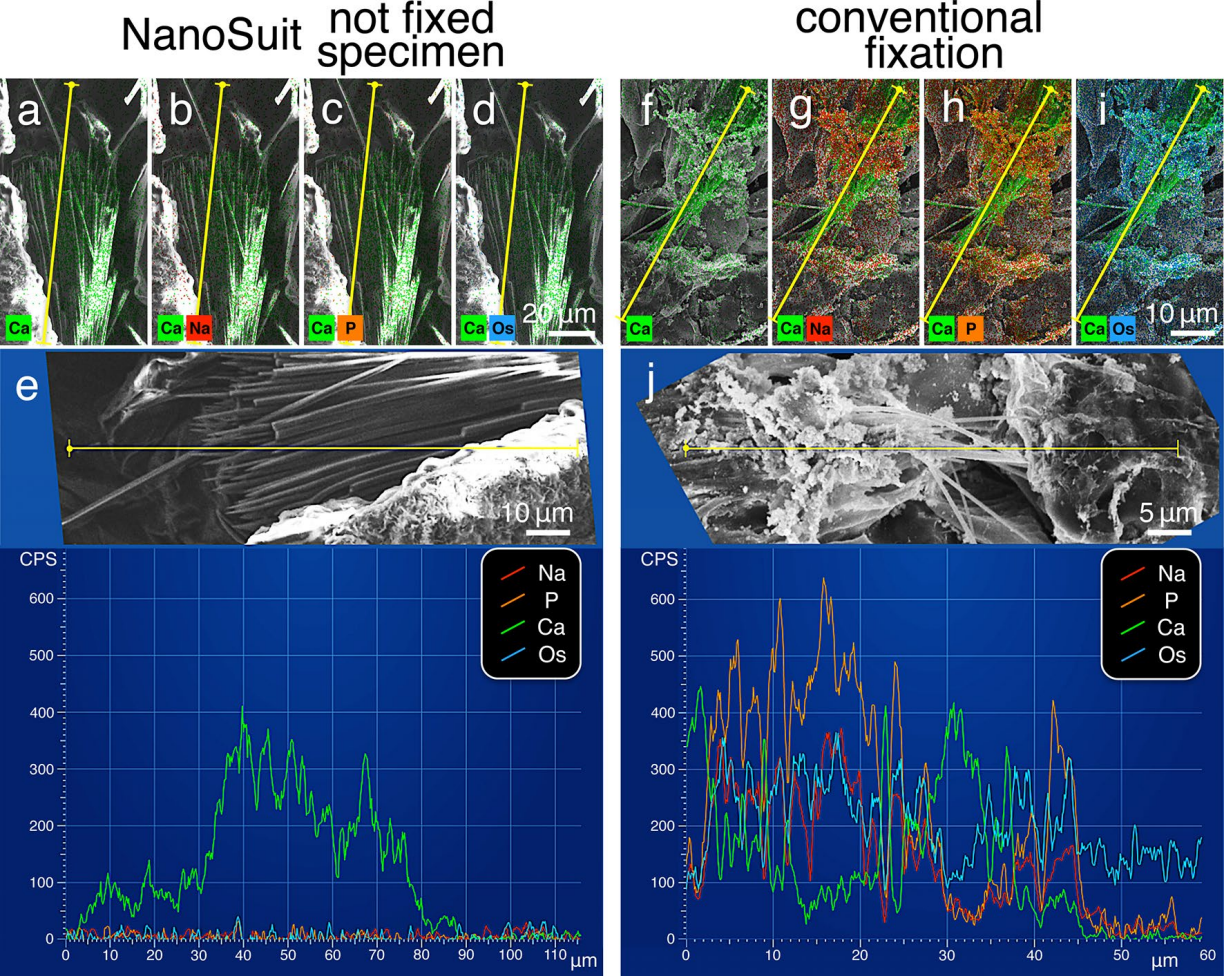
(**a**–**d**) Merged images of the EDS mapping analysis of the *Aloe* leaf slice prepared with the NanoSuit method and (**f**–**i**) by the conventional fixation method. The SEM image is merged with the elemental calcium (Ca; green) (**a**,**f**), sodium (Na; red) (**b**,**g**), phosphorus (P; orange) (**c**,**h**), and osmium elements (Os; light blue) (**d**,**i**) maps. (**e**,**j**) EDS line scans (along the yellow lines) in the same observation field (also shown as insets on these panels).

To further investigate the elemental composition on the microscale, we looked for additional elements present in the plant specimens. In the NanoSuit specimens, calcium was clearly observed in the needle-like crystals (Fig. 7a), while sodium (Fig. 7b), phosphorus (Fig. 7c), and osmium (Fig. 7d) were barely detected. The line analyses confirmed this result (Fig. 7e; Table 1). These results are consistent with the chemical composition of the *Aloe* gel, which contains low levels of sodium and phosphorus (Supplementary Fig. S2c). By comparison, the EDS analysis of the specimens prepared with the conventional fixation method revealed high levels of sodium (Fig. 7g,j), phosphorus (Fig. 7h,j), and osmium (Fig. 7i,j) over the entire surface of the specimens. These high values may be attributed to the phosphate buffer and osmium used in the conventional fixation method. In contrast to this result, the calcium counts in the crystal areas were similar to those of the NanoSuit specimen (Fig. 7f,j; Table 1).

We confirmed these results by analyzing the energy dispersive X-ray spectrum of the *Aloe* samples with NanoSuits and those that had been conventionally fixed (Supplementary Fig. S1d; Table 1). The average peak counts for sodium, osmium, phosphorus, and potassium were very much higher in the conventionally fixed specimens than in the NanoSuit samples (*P* < 0.01) (Supplementary Fig. S1d; Table 1). EDS analysis also showed that the average peak count for aluminium, which originated from the aluminium observation stub under the sample, was higher in the conventionally fixed preparations. This is presumed to be because of the fact that water was removed during the drying process in the conventionally fixed samples, and hence the signal from the stub easily penetrated the open or empty spaces in the samples, leading to such a high value. By comparison, the calcium counts were very similar in both samples (Supplementary Fig. S1d).

We also observed significant differences between the EDS analysis of the *Aloe* samples (Supplementary Fig. S1d) and *Drosophila* larvae (Supplementary Fig. S1c) prepared using conventional fixation methods. Specifically, the sodium, phosphorus, and osmium peaks were quite intense in the *Aloe* sample (*P* < 0.01) (Supplementary Fig. S1c,d; Table 1). These higher levels indicate that the *Aloe* tissue bonds with the buffer and fixative material far more strongly than *Drosophila* larvae, and suggest that the *Aloe* samples were strongly affected by the various reagents used in the sample preparation process. By contrast, the NanoSuit preparations result in similar levels of these elements in the *Aloe* and *Drosophila* samples, which are thus unaffected by this new preparation method. Hence, the combination of the NanoSuit and EDS techniques allows the detection of the chemical compositions of living biological samples, with low “background noise” compared with conventional fixation methods (Fig. 7e,j).

## Conclusions

The protective barrier formed on the surface of living tissues by plasma or electron beam irradiation—the NanoSuit—makes it possible to observe living tissue by conventional SEM without time-intensive pre-treatment procedures and causing damage to the specimens under high vacuum conditions. Our new results demonstrate that the NanoSuit method is not only helpful in obtaining high quality images but also enables us to study the in situ elemental compositions of fresh hydrous specimens at magnifications exceeding 10,000× using FE-SEM systems equipped with EDS capability. Such results cannot be achieved by using conventional fixation methods, and they have not been accomplished via Lv-SEM or E-SEM. Thus, the NanoSuit method with EDS and conventional SEM makes in situ elemental analysis possible for the first time for biological samples as well as other hydrated inorganic materials.

## Methods

### Experimental organisms

Third instar larvae (ca. 3 mm in body length) of the fruit fly *Drosophila melanogaster* (Oregon R variety) were used. To exclude any culture medium effects, they were washed at 24 ± 1 °C with distilled water several times and blotted briefly with a dry filter paper prior to the experiments.

*Aloe arborescens* was used as a plant specimen in this study. Specimens were cultured at room temperature and watered once a week. For SEM observations, ca. 1-mm-thick cross-sectional slices of leaf were cut with a scalpel.

### Microscopy

SEM observations were performed as described by Takehara et al. ^8^. FE-SEM was conducted using a JEM-7100F (JEOL) instrument operated at acceleration voltage of 1.0 kV. The vacuum level of the observation chamber was 10^–3^–10^–6^ Pa. In addition, the working distance was 8 mm, the aperture size *φ* was 100 µm, and the scan speed for each beam was 10–15 frames/s. The beam irradiation density was approximately 2.65 × 10^17^/m^2^ to 9.56 × 10^18^/m^2^, depending on the observation conditions. To record the dynamic movements of animals, imaging data from the SEM were directly transferred to a Hi-band digital formatted video recorder (SONY, BDZ-EW500).

### Energy dispersive X-ray spectroscopy (EDS)

Elemental analyses were carried out with a X-Max instrument (Oxford Instruments). Two EDS devices were equipped with SEM instrumentation that were operated at an acceleration voltage of 10.0 kV for the measurements. The EDS analysis was acquired using a 5 ms dwell time per pixel, hence the image acquisition speed was approximately 20 s per frame (the count rate was ~ 2,000 cps). To achieve high resolution, multistage scanning was conducted: for the EDS mapping analysis, six stages were moved, with each images requiring approximately 2 min in total for acquisition; for the EDS line analysis, three stages were acquired, taking approximately 1 min in total. The raw data was analyzed to obtain EDS spectra using the AZtec EDS Microanalysis software.

### Sample preparation for FE-SEM by the NanoSuit method

Samples were directly introduced into the SEM chamber without any conventional pre-treatments such as chemical fixation, dehydration, and ultrathin coating by electrically conducting materials. The ‘NanoSuit’ was formed immediately following irradiation by the electron beam (NanoSuit method)^3,7^. A low magnification (20–30×) electron beam was used to irradiate the entire surface of the sample, and the areas in which a NanoSuit formed were used for the subsequent SEM observations^8^.

### Preparation of samples using conventional fixation for scanning electron microscopy

Preparation for conventional SEM observations was performed as described by Takehara et al.^8^. Larvae and plant samples were prefixed with 2% glutaraldehyde in 0.1 M phosphate buffer (pH 7.4) and postfixed in 1% OsO_4_ in the same buffer. The samples were then dehydrated using a graded series of ethanol, transferred to t-butyl alcohol, freeze dried (JFD300, JEOL), and coated with an ultra-thin layer of OsO_4_ (PMC-5000, Meiwa).

### Plasma polymerization for fabricating biocompatible membranes

Preparation of insoluble plasma-irradiated membranes was performed as described by Takaku et al.^3^. Briefly, the metal-emitter from a standard ion-sputtering device (MSP-20-UM, VACUUM DEVICE) was removed, so that the plasma ions produced within the chamber were formed by the residual air-derived gas molecules.

To collect the ECS, 10 living larvae were placed on the center of a single glass slide. In a few minutes, ECS traces remained on the glass slide where the larvae had moved (Supplementary movie 1); whereas, the gel content within the *Aloe* samples was extracted from the cross-sectioned leaf slices and spread on a glass slide. Subsequently, the substances on the glass slides were polymerized by plasma irradiation. These specimens were irradiated at room temperature with plasma for 10 min at ca. 30 mA (+ 0.56 kV) under a vacuum level of ca. 10 Pa. The thickness of the membrane could be controlled both by the duration of irradiation time and the concentration of the solution.

### Statistical analysis

Statistical analysis was carried out using Student’s t test. The tests were two-tailed, and the actual *P* value for each test was generated with the significance level set at *P* < 0.01.

## Supplementary information

Supplementary Video 1

Supplementary Video 2

Supplementary Video 3

Supplementary Video 4

Supplementary Video 5

Supplementary Video 6

Supplementary Video 7

Supplementary file1
