# Supplementary material for: In situ elemental analyses of living biological specimens using ‘NanoSuit’ and EDS methods in FE-SEM
Source: Sci Rep. 2020 Sep 3;10:14574. doi: 10.1038/s41598-020-71523-8 (PMC7471950; doi:10.1038/s41598-020-71523-8)
Supplement: Supplementary file 8 — Supplementary file1 [file 41598_2020_71523_MOESM8_ESM.docx]

**SUPPORTING INFORMATION**

***In situ* elemental analyses of living biological specimens using ‘NanoSuit’ and EDS methods in FE-SEM**

Yasuharu Takaku^1^**^‡^***, Sayuri Takehara^1^**^‡^**, Chiaki Suzuki^1^**^‡^**, Hiroshi Suzuki^2^, Masatsugu Shimomura^3^ and Takahiko Hariyama^1^*

^1^ Preeminent Medical Photonics Education & Research Center, Institute for NanoSuit Research and

^2^ Department of Chemistry, Hamamatsu University School of Medicine, 1-20-1 Handayama, Higashi-ku, Hamamatsu 431-3192, Japan

^3^ Department of Bio- and Material Photonics, Chitose Institute of Science and Technology, 758-65 Chitose, Hokkaido 066-8655, Japan

‡ Y. T, S. T, and C. S equally contributed to this work.

*Corresponding Authors:

Takahiko Hariyama ([hariyama@hama-med.ac.jp](mailto:hariyama@hama-med.ac.jp))

Yasuharu Takaku ([ytakaku@hama-med.ac.jp](mailto:hariyama@hama-med.ac.jp))

**Table of Contents**

**Supplementary Figure S1.** Comparison of the EDS analysis on the selected specimen area.

**Supplementary Figure S2.** EDS line scan analysis for phosphate buffer used in the present experiments, the extra cellular substances (ECS) extracted from the surface of *Drosophila* larvae, and the gel extracted from the interior of an *Aloe* leaf.

**Supplementary movie 1.** ECS covering the surface of the larvae of *Drosophila*.

**Supplementary movie 2.** ECS biocompatible membrane in an aqueous solution. The self-standing plasma-irradiated membrane is water-insoluble. The membrane appears flexible when pipetted in water.

**Supplementary movie 3.** Semi-dried ECS substance in an aqueous solution. The substance not irradiated by plasma is water-soluble.

**Supplementary movie 4.** Movements of a living larva of *Drosophila* observed by SEM and the EDS analysis.

**Supplementary movie 5.** Gel contained inside of *Aloe*. The sticky substance is water-soluble.

**Supplementary movie 6.** Biocompatible membrane formed from gel of *Aloe* in an aqueous solution. The self-standing plasma-irradiated membrane is water-insoluble. The membrane shows flexibility, when it is pipetted in water.

**Supplementary movie 7.** Semi-dried gel of *Aloe* in an aqueous solution. The substance not irradiated by plasma is water-soluble.


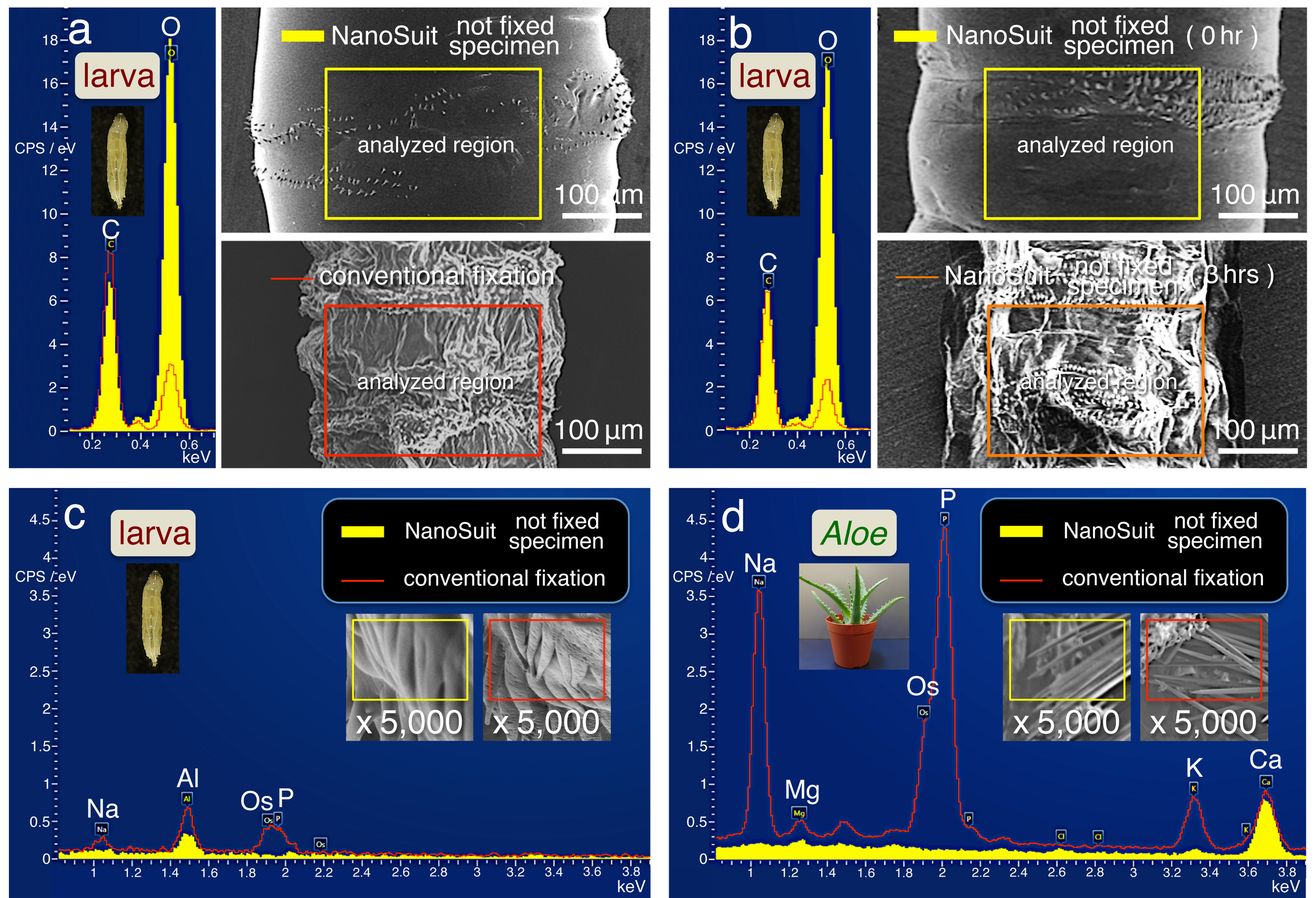


**Supplementary Figure S1.** Comparison of the EDS analysis on the selected specimen area (shown as rectangles overlaid on the insets). The elemental signals from samples prepared via two methods (NanoSuit and conventional fixation) of the *Drosophila* larvae (a, c) and the *Aloe* leaf slice (d). The change in the EDS spectrum between 0 and 3 hours is shown for a *Drosophila* larva prepared by the NanoSuit method (b).


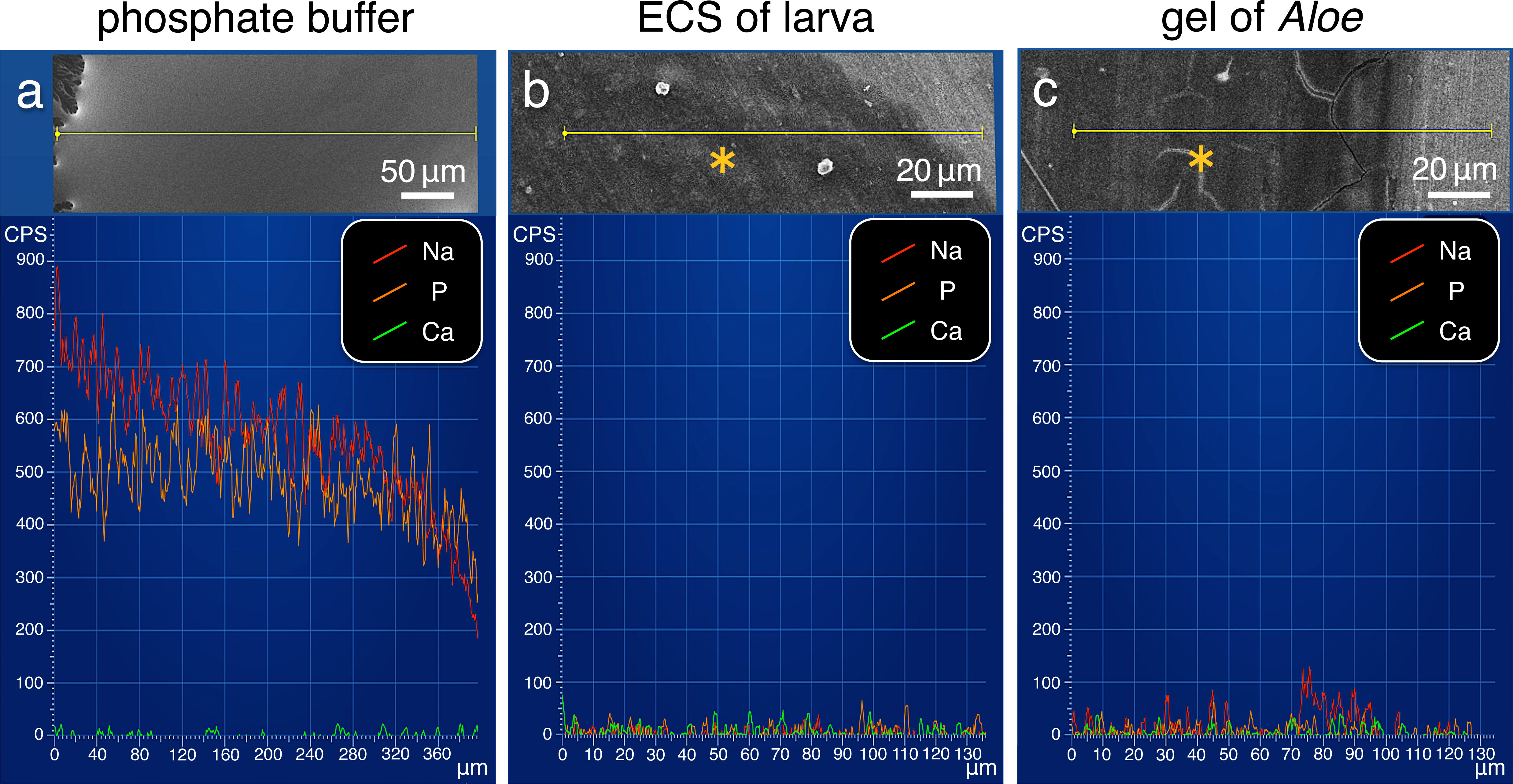


**Supplementary Figure S2.** EDS line scan analysis of the phosphate buffer used in the present experiments (a), the extra cellular substances (ECS) extracted from the surface of *Drosophila* larvae (b), and the gel extracted from the interior of an *Aloe* leaf (c). All samples were exposed to electron beam irradiation (see Methods). Asterisks in (b) and (c) indicate the positions of the ECS or gel on the observation stub, respectively (seen in dark gray). Yellow lines indicate the position of the line scan. Sodium (Na; red), phosphorus (P; orange), and calcium (Ca; green).
